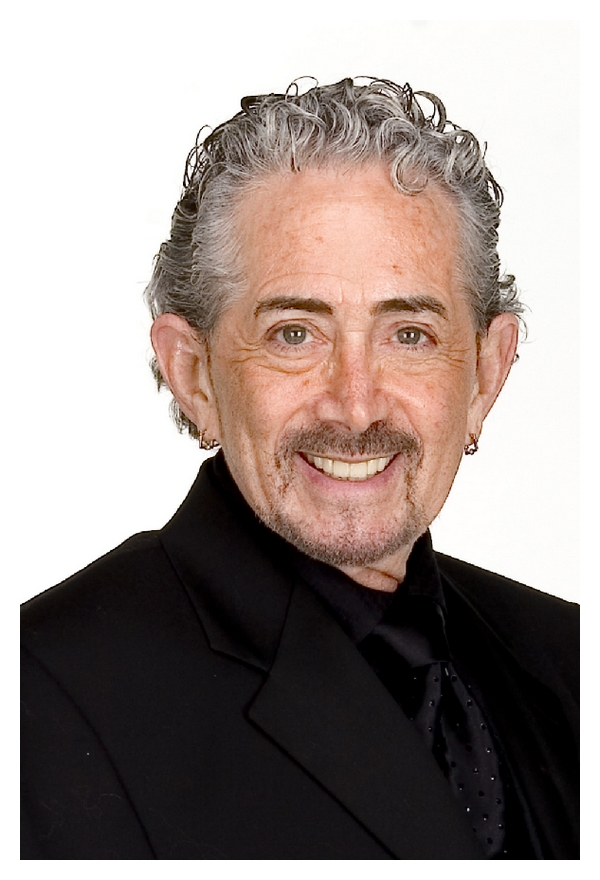# A Geriatric Patient: Age Is Not a Factor

**DOI:** 10.1155/2011/183471

**Published:** 2011-07-02

**Authors:** Lisa Klodnitskaya, Michele M. Harutunian, Santiago Mareque Buenos, Denise Estafan, Mark S. Wolff

**Affiliations:** ^1^Maimonides Hospital, Brooklyn, NY 11219, USA; ^2^Department of Cariology and Comprehensive Care, NYU College of Dentistry, New York, NY 10010, USA; ^3^Department of Periodontology, University of Santiago de Compostela, Santiago de Compostela, Spain; ^4^Department of Periodontology, Universidad de Catalana, Barcelona, Spain

## Abstract

*Objective.* A patient presented to the dental office expressing dissatisfaction with the appearance of his teeth, and as a result, of his smile. Our objective was to satisfy his initial chief complaint: “I don't like how my teeth look when I smile.” *Methodology.* Upon completing all initial exams and consultations, an esthetic dental treatment plan was formulated and agreed upon by both the practitioners and the patient. *Results.* The patient received periodontal treatment first to create esthetic gingival margins. Anterior ceramic crowns followed. *Conclusion.* The results surpassed all patient's expectations.

## 1. Introduction

Correction of pathological migration of anterior teeth can be a daunting and frustrating endeavor for any dentists. Different treatment alternatives are available to close the space between natural and artificial dentition. Such treatments range from simple composite restorations, periodontal therapy, veneers, and crowns to orthodontic realignment. 

This presentation is a patient who presented to our office expressing dissatisfaction with the gaps and color of his teeth, unhappy with the appearance of his smile. The objective of our treatment was to satisfy his initial complaint:“ I don't like how my teeth look when I smile.” Upon completion of the initial examinations and necessary specialty consultations, an esthetic treatment plan was formulated and agreed upon by both the practitioners and the patient. The patient initially received periodontal treatment to create esthetic gingival margins. Anterior porcelain crown fabrication followed. The esthetic outcome of therapy surpassed all patient expectations. 

## 2. Case Presentation

### 2.1. Chief Complaint

A 60-year-old male patient presented for a routine “dental check-up.” He immediately expressed his chief complaint: “I don't like how my teeth look when I smile.” He presented to our office with dental uncertainty and low self-esteem, a patient who never made eye contact while speaking softly and negatively about his unaesthetic smile.

**Figure 1:**
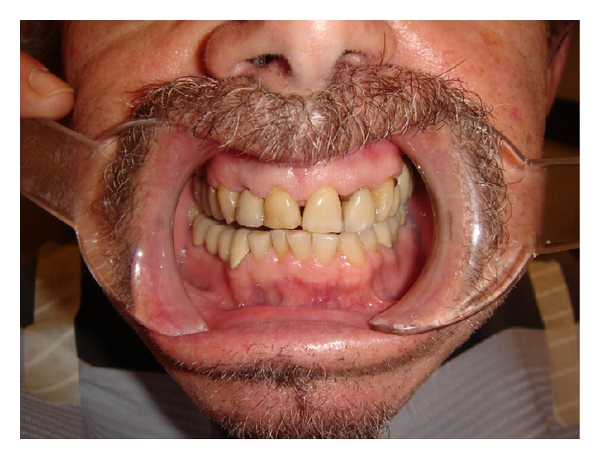


He presented with dark discolored teeth with clinical indications of occlusal wear. Immediately noticeable was the mismatch of his anterior crowns with his natural teeth, faulty, restorations throughout his mouth, and a reverse smile line (Figure 1). A reverse smile line is present as the incisal edges of the posterior teeth in the lateral portion of the smile are lower than those of the anterior teeth. The incisal edge contours of the anterior teeth do not follow the lower lip in the smile. He was anxious to address the unaesthetic appearance of his anterior teeth as soon as possible, and he also wanted to take advantage of this opportunity to close the spaces between his teeth and whiten his smile. 

## 3. Medical and Dental History

### 3.1. Findings

A comprehensive examination and review of the patient's medical history showed him to be in overall good health, with no abnormal findings. Nothing in his past medical history was relevant to any dental treatment. He was physically and socially active and sees his physician on regular basis.

His periodontal condition was fair but stable. He had few localized areas of gingival bleeding and supragingival plaque with mild calculus.

He had some missing teeth: multiple posterior teeth: number 1, 4, 13, 14, 16, 17, 19, 28, 29, and 32. 

He had new and recurrent carious lesions detected in teeth number 2, 6, 8, 9, 10, 11, and 21. 

He had implant-retained porcelain crowns on teeth number 13, 14, root canal therapy with post, core buildup and porcelain fused to metal crown on number 7 and ceramometal crown and fixed partial denture work in the following areas: number 3, 4, 5, 7, 10, 12, 18, 20, 27, 30, and 31.

## 4. Diagnosis and Planning

The patient's chief complaint and his desire for a more aesthetic smile was the driving force in formulation of this treatment plan. A thorough aesthetic examination was done, the patient's concerns were discussed, study models and preliminary photographs were taken, and radiographs were examined. The diagnosis was based on the patient's medical history, clinical and radiologic examination. 

Upon further evaluation, it was concluded that before any restorative or prosthodontic procedures were planned, a periodontal consultation was necessary to correct the unaesthetic gingival margin in the maxillary anterior region. 

 During the periodontal consultation, it was determined that the patient would require a crown lengthening procedure as well as a gingivectomy prior to any other treatment in order to respect the biological width and if ideal aesthetics were to be achieved. The patient would then receive porcelain crowns on his six anterior teeth.

 It was noted that the patient had many interproximal carious lesions. He received a caries risk assessment, extensive oral hygiene instructions, and a prescription for Prevident 5000 toothpaste. Following an adult prophylaxis and some selective hand scaling, a shade of his teeth was taken. The shade of his teeth ranged from A3 to A4 using the Vita Lumin (Vident, Germany) shade guide. The patient desired to have a lighter shade, and shade A2 was agreed upon.

He also had poorly matched composite and porcelain restorations, uneven gingival margins, black interproximal triangles, and severe occlusal wear, with many chipped incisal edges (Figure 1). 

Treatment options were explored and in order to address all of the patient's requests, as well as to satisfy all clinical requirements, the following treatment plan/sequence was developed for and accepted by the patient:

- performing surgical crown lengthening and gingivectomy in area number 6 through number 11,
- Postoperatory followup: suture removal in 10–14 days, and 8 weeks of healing, were allowed,
- performing all operative restorations on teeth: number 2 MOD, number 20 B, number 21 MOD and direct composites restorations,
- Preparing teeth number 6, 7, 8, 9, 10, and 11 and provisionalizing (Luxatemp, Zenith/DMG; Englewood, NJ),
- refining prepared teeth [1] and taking impressions for Procera-AllCeram crowns (Nobel Biocare Sweden),
- inserting crowns with FujiCem, a resin-modified glass ionomer [2],
- follow-up treatment with hygiene and home care for optimal gingival health and high caries risk.

## 5. Clinical Protocol

### 5.1. Surgical Procedure

To prepare for the execution of the treatment plan, first a simulated gingivoplasty was performed on the patient cast, followed by a diagnostic wax-up. This procedure was performed so that the height of the gingival margins of the central incisors would be higher than that of the laterals and be even with the canines. 

The mock surgery and diagnostic wax-up were followed to help better visualize the outcome and allow for the fabrication of the periodontal surgical guide and for provisional. The surgical guide was fabricated utilizing a clear vacuform matrix. 

**Figure 2:**
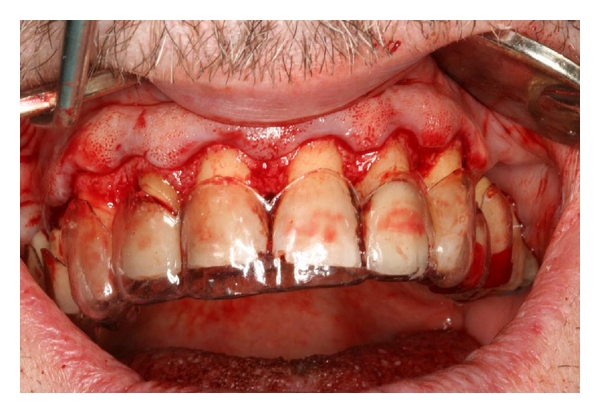


 The patient received infiltration local anaesthesia extending from tooth number 5 to number 12 area utilizing 2% lidocaine with 1 : 100,000 epinephrine. Intrasulcular incisions were made along the gingival margin from tooth number 5 to number 12, and a 1 mm to 2 mm submarginal incision in the palatal region to expose previous prosthetic margins wherever FPDs were present, in order to facilitate restorative treatment. Interdental papillary tissues were preserved as much as possible during incisions and flap elevation in order to avoid compromising the papillary height. No vertical release incisions were utilized. Full-thickness facial and lingual flaps were elevated to expose the underlying bone. The facial tissue was elevated beyond the mucogingival attachment. Ostectomies and osteoplasties were performed with a high-speed rotary instrument and, number 2 and number 4 carbide round burs were used as dictated by the surgical guide. A distance of 3 mm was measured from the desired gingival margin, determined by the stent (Figure 2), to the crest of bone, in order to allow for the biologic width and sulcus to be reestablished [3]. No interproximal bone was removed. Flaps were repositioned, and primary closure was achieved with interrupted and vertical mattress sutures, as described by a modification of the “curtain procedure” [4], in order to avoid shrinkage of the interdental tissues as much as possible. 5/0 Vicryl sutures (Ethicon, Midwest Dental) were used. 

**Figure 3:**
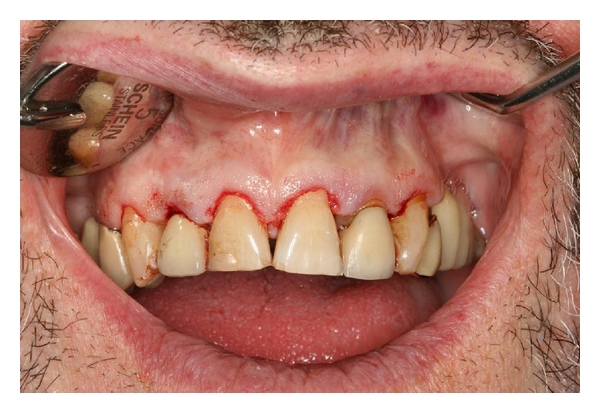


The patient was instructed to rinse twice a day for 14 days with a 0.12% chlorhexidine rinse and to avoid brushing the area. Ibuprofen 800 mg was prescribed for pain, 3 times a day for 5 days. Sutures were removed 14 days postsurgery. The patient received hygiene instructions, and soft tissue was permitted to heal for 8 weeks to allow for soft tissue contraction, and a secondary procedure was scheduled in order to refine the level of the gingival margins precisely [5] following the contours of the wax-up by means of the same surgical guide. Local anaesthesia 2% lidocaine with 1 : 100,000 epinephrine was used in the areas of teeth number 5 to number 12, and an internal bevel gingivectomy was carried out in all areas necessary to achieve the desired gingival margin level (Figure 3).

## 6. Restorative Treatment

Four weeks following the gingivectomy procedure, the patient was scheduled to begin restorative work. At the first appointment we planned to prepare the patients teeth, make final impressions, and provide interim restorations utilizing the diagnostic models. The patient was anesthetized with 2% lidocaine with epinephrine 1 : 100,000 by infiltration. The porcelain fused to metal crown was removed from tooth number 7, a cast post was noted beneath the crown. Teeth number 6, 7, 8, 9, 10, and 11 were prepared for all ceramic crowns. The interproximal carious lesions were completely removed, and contours were restored with bonded composite resin where necessary. The teeth were reduced approximately 1.3 mm–1.4 mm [6] on the facial, interproximal, and lingual surfaces, and a 2 mm incisal reduction was ensured to provide minimum material thickness. The cast post number 7 was slightly reduced on the facial surface to allow for proper thickness of the aluminum oxide ceramic coping and overlying porcelain [7]. Coarse chamfer bur (number 30006-144) and fine chamfer bur (number 38006-145 (Brassler, USA)) were utilized to achieve the preparation and create chamfer margins. In order to make a final impression, retraction cord number 00 (Ultradent Products Inc.,) was carefully placed in the gingival sulcus exposing 0.5 mm of uncut tooth structure. Final impressions were taken with Reprosil Quixx Putty, Vinyl Polysiloxane Impression Material Type 1, Very High Viscosity, and Reprosil Regular Body Hydrophilic Vinyl Polysiloxane Impression Material Type 1, Medium Viscosity. Provisional crowns were fabricated using Luxatemp (Zenith/DMG Luxatemp Fluorescence). Luxatemp was flowed into an alginate impression of the diagnostic wax-up and seated into the patient's mouth. After about ninety seconds, the material had partially hardened and was removed from the prepared teeth. The temporary was removed, and margins were finished with a Flame Shape Bur (number 888EF012 (Brassler, USA)) to avoid overhangs and ensure gingival health. The interproximal areas were easily accessible to the patient for proper oral hygiene. The interim crowns were then temporarily cemented utilizing temporary cement (TempBond). The desired shade (A2), shape, and contour were detailed in the laboratory prescription, and digital photographs were sent to the laboratory as well.

## 7. Final Restoration Placement

Prior to the patient visit, all the ceramic crowns were first tried in on the cast for verification that their contours had followed the diagnostic design. Anesthesia was achieved, temporary crowns were removed and the porcelain restoration was tried in. The teeth were cleaned with course pumice and water to remove residual temporary cement and debris. The porcelain crowns were tried in one by one to assure appropriate margins and occlusion and then shown to the patient. Upon patient approval, photographs were taken and preparations for final cementation began. The rubber dam was placed on the patient. When the teeth were ready for cementation, the crowns were prepared for cementation [1]. The cement of choice was FujiCEM (GC America), a resin-modified glass ionomer cement [2]. This cement was chosen as a definitive luting material due to its stability, lack of thermal expansion, and minimal microleakage [8]. The interior surfaces of each porcelain crown was coated with a thin layer (less than 1 mm) of the cement [9]. The crowns were seated one by one by gently rocking them into place and using finger pressure. Occlusion and proper seating were confirmed, and pressure was maintained until the luting cement hardened into a gel-like consistency. Excess cement was removed, utilizing a brush (Microbrush International 2 mm) facially and lingually, and floss was utilized in the interproximal regions. The rubber dam was removed and the occlusion was checked for light centric contact and proper contact on lateral excursion. Adjustments were made as necessary and porcelain repolished [10]. A follow-up appointment was scheduled for a week later.

Literature states that the average crown can last 7–10 years [11].

## 8. Conclusion

**Figure 4:**
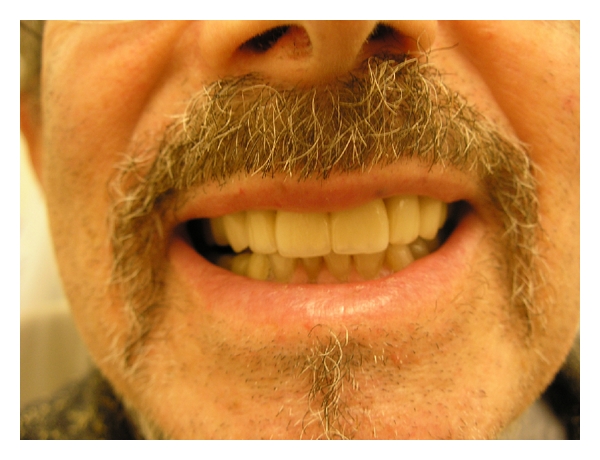


**Figure 5:**
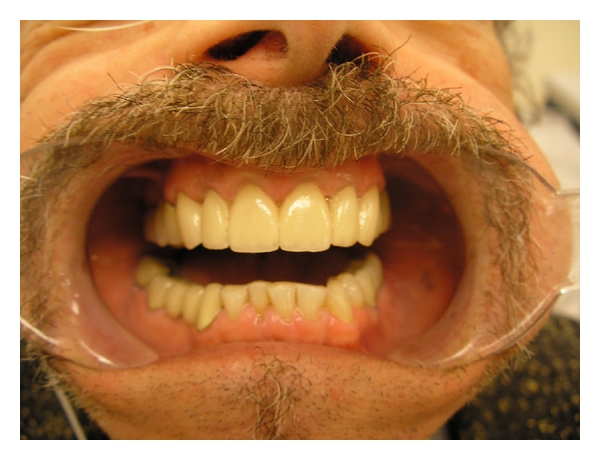


**Figure 6:**
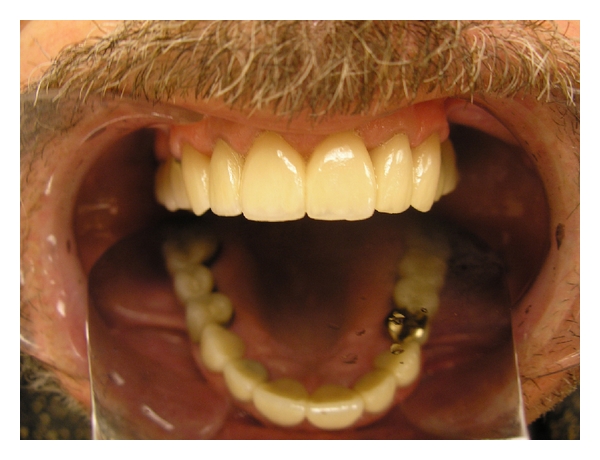


At the follow-up appointment, the patient was very happy with the esthetic result and appeared to be in great spirits. He was pleasantly surprised by how his smile had been transformed (Figure 4) from a worn, aged, dark, and reversed smile, to a bright, symmetrical, natural look (Figures 5 and 6). Following this procedure, his career, which requires him to appear in the public, has taken off immensely. He used to appear on television and in print, so smiling was a necessary requirement, which he could not fulfill before. His aesthetically pleasing new look has increased his confidence, and enhanced his career (Figure 7). He left our office with his head held high and a big smile on his face. An aesthetic smile instills confidence, can improve interpersonal relationships, and make a person happier overall [12].

## Figures and Tables

**Figure 7:**